# On Cholera

**Published:** 1849-08

**Authors:** Reid Clanny


### PATHOLOGY AND PRACTICE OF MEDICINE.

*On Cholera.* By Dr. Reid Clanny.—Eighteen years have elapsed since it was my painful duty to report to Government the outbreak of cholera in England, and that Report formed the leading article in each of the London newspapers of the day. I little expected to witness the return of the pestilence amongst us, though I held in remembrance the observation of the late Sir Henry Halford—that the cholera would return, as has been experienced in respect to small-pox, &c.

At this moment I am reminded of the words of Ovid,—"Dum vires annique sinunt tolerate laborem."

From experiments and observations made during the first and second visitations of *the* cholera in this town, I have come to the conclusion, that its origin and persistence in each individual case may be attributed to the abnormal state of endosmose and exosmose in the lungs. In the year 1834, my experiments on human blood satisfied, I believe, most of my professional brethren, that atmospherical air was received continuously through the air-cells and pulmonary veins into the blood, by endosmose and that portion of the oxygen which was not used in the formation of carbonic acid in the blood-vessels, was by exosmose conveyed again into the atmosphere with the halitus of the air-cells of the lungs. The nitrogen also was, in the process of circulation, to a certain degree, diminished in quantity. These observations will, *in limine*, suffice. We are aware, that when the effects of nature are too intricate, experiments are the only *media* which remain for us in our investigations. Whether the magnetic state of the atmosphere be the exciting cause of this abnormal state of the air-cells of the lungs, I am unable to say; but I am strongly inclined to be favorable to that opinion, from the report in the *Medical Times* for the 7th of last month. I regret that I was so much engaged during the last visitation of the cholera, that, on this point, I made no experiments.

We are aware, that, though different descriptions of gases pass through membranous *media*, a remarkable difference is observable in the relative rapidity of transmission. This fact has been accurately investigated by the experiments of Drs. Faust and Mitchell. They transmitted gases through membranes which were more dense than those of the lungs. (*Vide American Journal of Medical Science*, No.13. *Vide etiam* Milne Edwards' valuable experiments.) We are aware, and the general impression is, that we cannot trace any nervous filaments to the fibres of cellular tissues, though such threads may be seen passing between them to the neighboring organs. The insensibility in its healthy state seems also to indicate the absence of nerves; but as pain is experienced during inflammation, we must admit the existence of some communication with the sensorium commune. Respiration is effected by the contact of the atmospherical air with blood, the smallest particles of which, in its passage through the innumerable capillaries which ramify on the air cells of the lungs, are, by means of the immense surface, (which all these cells offer,) exposed to the action of the atmospherical air. The chemical process which ensues between the air and the blood is carried on through the delicate membranous parietes of the cells, and in accordance with the laws of endosmose and exosmose.

The respiration is everything, and may be considered as the *primum mobile* of the whole animal system. When atmospherical air is received into the air-cells of the lungs, a portion of it comes into union with the free carbon of the blood, in the blood-vessels, forming carbonic acid in equal volume with the oxygen gas; from this process the arterial blood owes its property of being the sole stimulus of living structures; venal blood which has not undergone this change has a poisonous action on the organs of the body, particularly on the nervous system, overcoming their irritability; hence, should the air-cells of the lungs be so acted upon by the magnetism or electricity of the atmosphere, the functions of the body are, in some instances, in a few minutes, suspended. Hence the symptoms of cholera of rapid type. Hence the sudden deaths from cholera upon its first invasion of a town or even district. In one man, says an eye-witness, (p. 50, Madras Reports,) the prostration of strength was so great, that he could hardly move a limb, though he had been, but fifteen minutes before, in perfect health, and actively employed in his business of a gardener. "As an instance," says another, "a Lascar, in the service of an officer, was seized in the act of packing up rice, previous to going out to cut grass close to his master's tent; and, being unable to call for assistance, he took up small stones and pitched them towards him, (the eye-witness,) in order to attract his notice. This man died in an hour." Should the air-cells of the lungs refuse to receive the atmospherical air, especially the oxygen thereof, we would expect *à priori*, to find that individuals so placed would experience, in the first instance, great debility, hindered respiration, and ultimately extinction of that most important function, and death. During this progress, should the individual survive the first impulse, universal debility takes place—vomiting, purging, and their concomitants,—vomiting to excess, even of blood,—purging to excess, even of blood, as we have all witnessed, more or less,—cramps, from excessive debility of the stomach and bowels,—thirst, cold, clammy skin, suffused, half-closed eyes, and weakened intellect, are forerunners of death.

As only a certain portion of oxygen, from the state of the atmosphere, finds its way into the blood, hence a gradual precipitation.—*Lond. Med. Times.*

